# A Case of Myxoid Malignant Peripheral Nerve Sheath Tumor in a Patient With Carney Complex

**DOI:** 10.1155/crip/4337436

**Published:** 2025-07-31

**Authors:** Kazuhiro Kobayashi, Natsuko Suzui, Hirofumi Shibata, Takenori Ogawa, Tatsuhiko Miyazaki

**Affiliations:** ^1^Department of Pathology, Gifu University Hospital, Gifu, Japan; ^2^Department of Otolaryngology-Head and Neck Surgery, Gifu University Graduate School of Medicine, Gifu, Japan

**Keywords:** Carney complex, MPNST, PRKAR1A mutation

## Abstract

**Background:** Carney complex (CNC) is a group of disorders characterized by endocrine hyperactivity or tumors, abnormal skin pigmentation, myxomas of the skin and heart, and adrenocortical and pituitary tumors; in most cases, the disorder is inherited in an autosomal dominant manner.

**Case Presentation:** We, herein, report a female patient who had undergone a total of seven left parotid tumor resections since the age of 45 years. At age 50, genetic testing confirmed a c.597del C (p. Phe200LeufsX6) mutation in the type-1*α* regulatory subunit of cAMP-dependent protein kinase (PRKAR1A); this led to a diagnosis of CNC for the patient and the patient's second and third daughters. At the age of 55, the left parotid gland became rapidly enlarged, and surgery was performed because recurrence was suspected. Intraoperative rapid pathological diagnosis revealed a mucous tumor with an unknown differentiation grade; considering the possibility of malignancy, resection was thus performed. The specimen from the first surgery at our hospital contained an S100^+^, CD34^+^ mucinous spindle cell tumor. During follow up, the patient was treated as a case of atypical myxoid tumor with low-grade malignancy. Due to recurrence at 60 years old, surgery was performed. The tumor was sheet- and cord-like, partially saccular, and had a cribriform pattern of spindle-shaped or linear atypical cells; the stroma contained abundant myxomatous matrix deposits. Approximately 1% of the tumor cells were S100^+^, CD34^+^, SOX10^+^, and MIB-1 positive, and the growth was diagnosed as a myxoid low-grade malignant peripheral nerve sheath tumor (MPNST).

**Conclusion:** We believe this is the first report of a CNC patient developing a myxoid MPNST derived from the salivary glands.

## 1. Introduction

Carney complex (CNC) is a group of disorders characterized by endocrine hyperactivity or tumors, abnormal skin pigmentation, myxomas of the skin and heart, and adrenocortical and pituitary tumors; in most cases, the disorder is inherited in an autosomal dominant manner. Mutations in the type-1*α* regulatory subunit of cAMP-dependent protein kinase (PRKAR1A) are implicated in the development of CNC.

MPNSTs typically affect adults with a mean age of 50.7 and a male-to-female ratio of 1.5:1. The prevalence for males has been reported in both sporadic and NF1-associated MPNST contexts. Head and neck MPNSTs have been reported in various sites, such as the cheek, tongue, infratemporal fossa, brachial plexus, paranasal sinus, orbit, and parotid gland [1]. However, primary intraosseous tumors are extremely rare, with only a few cases documented in the English-language literature [2–7].

Here, we report a case of CNC diagnosis in a mother and two of her daughters; the mother developed a myxoid MPNST derived from the salivary glands, which to our knowledge has not previously been reported.

## 2. Case Presentation

### 2.1. Medical History

Left parotid tumor resection, pituitary tumor removal (GHoma [growth hormone–producing pituitary adenoma]), laparoscopic salpingo-oophorectomy (serous cystadenoma), and multiple cardiac tumor resections (myxoma).

### 2.2. Current Medical History

**Figure 1:**
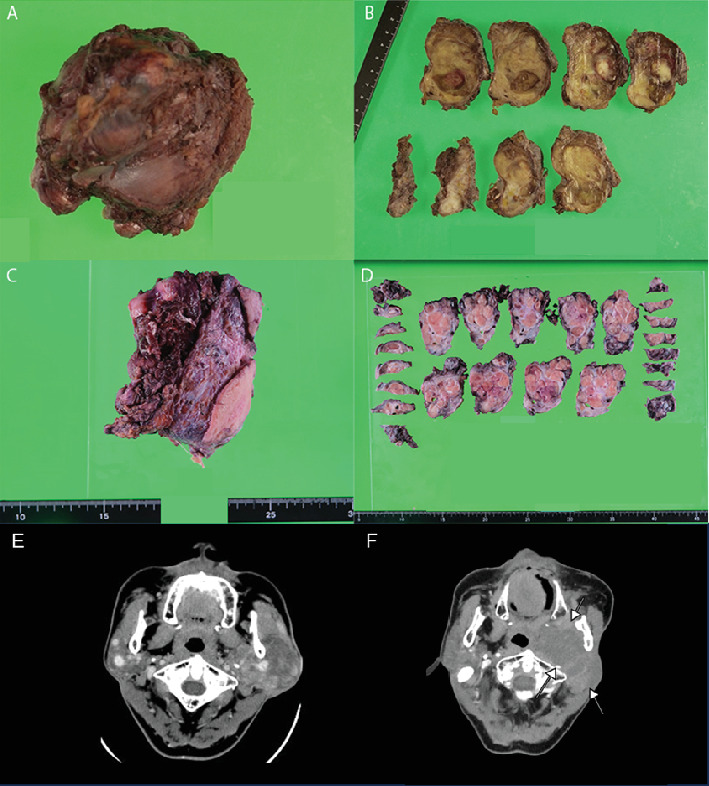
(A) Macroscopic image of the first surgical specimen performed at our hospital when the patient was 55 years old. (B) 7 cm × 4.5 cm yellow, well-defined lobulated mass. White areas were mixed inside the mass. (C) Macroscopic image obtained at the surgery when the patient was 68. (D) A yellow lobulated mass measuring 7 cm × 5 cm was seen. The tumor invaded the surrounding muscle tissue. (E) Preoperative contrast CT image of a 55-year-old: The left parotid gland is occupied by a massive tumor measuring 60∗50 mm. Most of the tumor shows contrast enhancement, but it is heterogeneous, with nodular enhanced areas recognized from the early phase of contrast, especially around the peripheral part. (F) Preoperative CT image of a 68-year-old: postleft parotid gland tumor removal. The recurrent tumor extends from the gland to the parapharyngeal space, reaching the submandibular area.

The female patient has undergone a total of seven times of left parotid tumor resections since the age of 45 years. At age 45, she underwent surgery for a left parotid tumor at another hospital. At that time, she was diagnosed with a benign tumor (details unknown). At the age of 50, genetic testing of the recurrent tumor confirmed a c.597del C (p. Phe200LeufsX6) mutation in the PRKAR1A; the patient and the patient's second and third daughters were diagnosed with CNC [8, 9]. At the age of 55, the residual left parotid gland rapidly enlarged, and surgery was performed. Intraoperative rapid pathological diagnosis revealed a mucinous tumor with an unknown differentiation grade; tumor was resected at our hospital. The resected tumor at that time manifested S100^+^, CD34^+^ mucinous spindle cell tumor (Figures 1A, 1B, and 1E). Although we considered the lesion of myoepithelioma or solitary fibrous tumor (SFT), neither was considered typical in this case. Due to metastasis to the lymph node and involvement of the submandibular gland, the patient was followed up as having a low-grade malignant atypical myxoid tumor. At age 60, surgical intervention for a tumor recurrence revealed invasion of the temporal muscle, with a malignancy of the same histologic type as the previously resected. After that, recurrences were seen in the lymph node and parapharyngeal stromal at age 63, in the parotid gland at age 65, and a parapharyngeal space at age 68 with the same pathological manifestations. The patient died at 73 years of age due to systemic metastasis of the tumor.

### 2.3. Family History

Family history is as follows: The second daughter was diagnosed with CNC, had facial and lip pigmentation, and underwent laparoscopic bilateral adrenalectomy. The third daughter was also diagnosed with CNC, had facial and lip pigmentation, and underwent laparoscopic bilateral adrenalectomy. She was also found to have multiple myxomas in the left atrium.

### 2.4. Gross Pathology

A 7 cm × 5 cm yellow lobulated mass was seen. The tumor invaded the surrounding muscle tissue (Figures 1C, 1D, and 1F).

### 2.5. Histological Findings

**Figure 2:**
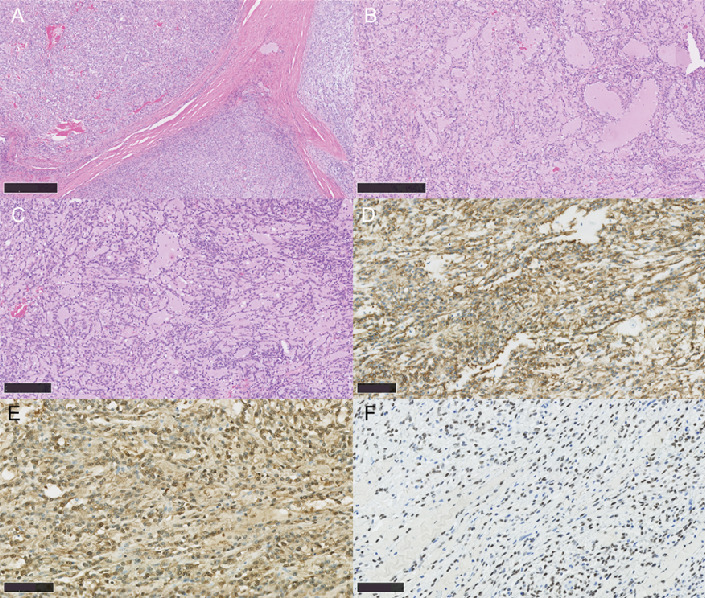
(A) Tumor with a well-defined fibrous capsule and a sheet-like, cord-like, partially saccular, cribriform pattern was also seen in some places (scale bar: 500 *μ*m). (B) Microcystic areas where cells showed tubular structures were also seen (scale bar: 500 *μ*m). (C) Proliferation of spindle-shaped cells with bilobed cytoplasm was noted. Pale eosinophilic myxomatous matrix was observed between the cells (scale bar: 250 *μ*m). (D) CD34 was diffusely positive (scale bar: 100 *μ*m). (E) S100 was diffusely positive in the nuclei and cytoplasm (scale bar: 100 *μ*m). (F) SOX10 was diffusely positive in the nuclei (scale bar: 100 *μ*m).

Tumor cells were uniformly and somewhat sparsely distributed, and the lesions were generally nodular with a fibrous capsule surrounding them, but some had invasion into the surrounding area. The interior showed fibrous septa. The growth of spindle-shaped or linear atypical cells in a sheet-like, cord-like, partially saccular, or cribriform pattern was observed, with abundant myxomatous matrix deposition in the stroma. The atypical cells had a high nuclear/cytoplasm ratio: nuclei with nucleoli, a well-defined nuclear border, and increased fine chromatin. The cytoplasm was predominantly acidophilic but was accompanied by granular basophilic structures in some places. Infiltration of neutrophils, lymphocytes, and plasma cells was observed. The stroma was partially accompanied by atrophied salivary gland tissue. The tumor had also invaded the surrounding musculature and adipose tissues. Immunohistochemistry showed that tumor cells were S100^+^, CD34^+^, SOX10^+^, c-kit^−^, desmin^−^, DOG1^−^, ER^−^, HMB45^−^, STAT6^−^, Oct4^−^, synaptophysin^−^, AE1/AE3^−^, SMA^−^, and vimentin^+^; for a small fraction of cells, EMA^+^, synaptophysin^−^, BCL2^−^, and *β*-catenin^−^ and MIB-1 positivity was about 1% (Figure 2 and Table 1). Based on these results, this case was diagnosed as myxoid low-grade MPNST.

## 3. Discussion

CNC is a disease characterized by the development of skin pigmentation, endocrine hyperactivity, adrenocortical tumors, pituitary adenomas, and cardiac myxomas [10, 11]. Currently, CNC is diagnosed according to the diagnostic criteria listed in Table 2. According to Stratakis et al., the diagnostic criteria for CNC require either two or more major manifestations—such as spotty skin pigmentation, cardiac myxoma, or endocrine tumors—or one major manifestation along with a supplemental criterion, such as a PRKAR1A mutation or an affected first-degree relative. These criteria are widely accepted and have been used in the evaluation of this patient [12]. In the case presented here, about half of the cardiac myxomas and CNCs classified in the major criteria are inherited in an autosomal manifest manner. While 70% of patients diagnosed with CNC are known to be hereditary, about 30% are considered to be nonhereditary mutations. CNC is known to be complicated by a variety of tumors and symptoms.

Furthermore, rare manifestations of CNC continue to be reported, expanding the clinical spectrum of the disease. For example, Savva et al. described a case of bilateral myxoid fibroadenomas of the breast in a CNC patient, emphasizing the variability in tumor presentation and the need for more comprehensive surveillance in affected individuals [13]. Such cases underscore the importance of accumulating clinical data to better define the natural history of CNC and improve diagnostic strategies.

Our survey of the CNC literature up to 2022 revealed only limited reports of malignancies; these included thyroid cancer, acinar cell carcinoma and adenocarcinoma of the pancreas, and colonic and gastric carcinoma (Table 3). Reports of myxoid malignant soft-tissue tumors are minimal. Psammomatous melanotic schwannoma (PMS) is a rare tumor of the central and peripheral nervous system that occurs in some CNC patients [14, 15]; the tumor is multicentric with frequent calcification and pigmentation [16, 17]. The most frequent sites of PMS are the gastrointestinal tract and paraspinal sympathetic chain [18]. Spinal cord tumors occur in adults (average age 32 years) and generally manifest as painful lesions with nerve root involvement. Approximately 10% of CNC-associated PMS go on to become malignant [19]. This tumor was renamed “malignant melanotic nerve sheath tumor” in the 5th edition of the *WHO Classification of Tumors of Soft Tissue and Bone* [20]. In our present case, there was no melanin deposition or sandy calcification deposits, which are considered characteristic of PMS. Therefore, we considered the tumor to be a malignancy of the nervous system that had not been previously reported to occur in association with CNC. In addition, we believe that this tumor should be categorized as a myxoid MPNST because it recurred locally and metastasized to the lung.

More than 70% of patients diagnosed with CNC carry mutations in the PRKAR1A gene (the “CNC1 locus”); the frequency increases to 80% in patients with Cushing syndrome due to primary pigmented nodular adrenocortical disease (PPNAD) [21, 22].

To date, 401 unrelated CNC families of diverse ethnic origins have been reported [21, 23, 24]. Pathogenic mutations in PRKAR1A include single nucleotide substitutions, small (≤ 15 bp) deletions/insertions, complex reorganizations that span the entire open reading frame of the gene, and large deletions that cover most exons [15]. Most of these mutations are unique to a single family, with only three pathological variants (c.82C>T, c.491_492delTG, and c.709-2_709-7 delATTTTT) identified in more than three unrelated families [15, 21, 25].

The PRKAR1A mutations associated with CNC lead to loss of function of the R subunit, which normally inhibits the catalytic C subunit of PKA. The activity of the “uninhibited” C subunit leads to increased phosphorylation of the downstream PKA substrate, cAMP response–binding protein (CREB), increased cell proliferation in cAMP-responsive tissues, and tumor formation in CNC-affected tissues [26, 27].

It is challenging to predict the molecular pathological mechanisms that directly link CNC and MPNST due to the absence of case reports connecting them directly. However, it is hypothesized that Protein Kinase A (PKA) typically acts to suppress the MAPK pathway in normal cells. In cells with PRKAR1A mutations, where regulatory subunit Type 1A is either absent or the PRKAR1A mutation is ineffective, an overproduction of other regulatory subunits (mainly Type II PKA) or a defect in Type I PKA may occur. These changes in CNC cells could relatively activate the extracellular signal-regulated kinase in the MAPK pathway, potentially leading to abnormal proliferation and growth.

The development of MPNST has been reported to involve the activation of a part of the classical MAPK pathway, the RAS-RAF-MEK pathway [28]. These mechanisms may have caused the development of MPNST in this patient. However, many aspects, such as malignancy, cannot be explained by these mechanisms alone, and further examination is awaited.

In a recurrent tumor in the parapharyngeal space of the patient discussed in our report, ARID1A G2087R, PRKR1A F200fs∗6, and RAD21 amplification were identified as disease-relevant alterations by genetic testing. PRKAR1A gene mutations are included among the supplemental criteria used to make a diagnosis of CNC (Table 1). How the PRKAR1A mutation detected in this CNC is related to tumorigenesis is currently unclear.

*Prkar1a* +/− mice develop nonpigmented schwannomas and fibrotic bone lesions beginning at about 6 months of age; 10% of the mice subsequently develop thyroid tumors [29]. Another mouse model with significantly more *Prkar1a* downregulation and significantly higher cAMP signaling produced a more severe CNC phenotype [30]. Although reported in *Prkar1a* heterozygous mice, the presence of nonpigmented schwannomas is consistent with our conclusion that the cancer is a mucinous malignant neurogenic tumor arising in association with CNC. Careful clinical observation and documentation of future CNC patients will help to substantiate our claims.

## 4. Conclusion

We have identified a previously unreported myxoid malignant nervous system tumor that developed in a patient with CNC.

## Figures and Tables

**Table 1:** Immunohistochemistry of the tumor.

**Immunohistochemistry**	
S100	+
CD34	+
c-kit	−
Desmin	−
DOG1	−
ER	−
HMB45	−
STAT6	−
SOX10	+
OCT-4	−
Synaptophysin	−
AE1/AE3	−
SMA	−
Vimentin	+
EMA	(Partially)+
BCL2	−
*β*-Catenin	−
Ki-67 labeling index	1%

**Table 2:** Diagnosis criteria for CNC. ⁣^∗^To make the diagnosis of CNC, a patient must either (1) exhibit two of the major criteria confirmed by histology, imaging, or biochemical testing or meet (2) one major criterion and ⁣^∗∗^ one supplemental criterion with histologic confirmation.

**Diagnostic criteria for CNC**	
Major criteria	1. Spotty skin pigmentation with typical distribution (lips, conjunctiva and inner or outer canthi, vaginal, and penile mucosa)
	2. Myxoma⁣^∗^ (cutaneous and mucosal or cardiac myxoma)
	3. Breast myxomatosis⁣^∗∗^ or fat-suppressed magnetic resonance imaging findings suggestive of this diagnosis
	4. PPNAD⁣^∗^ or paradoxical positive response of urinary glucocorticoid excretion to dexamethasone administration during Liddle's test
	5. Acromegaly as a result of growth hormone (GH)–producing adenoma
	6. LCCSCT⁣^∗^ or characteristic calcification on testicular ultrasound
	7. Thyroid carcinoma⁣^∗^ (at any age) or multiple hypoechoic nodules on thyroid ultrasound in prepubertal child
	8. Psammomatous melanotic schwannomas (PMSs)⁣^∗∗^
	9. Blue nevus, epithelioid blue nevus (multiple)⁣^∗∗^
	10. Breast ductal adenoma (multiple)⁣^∗∗^
	11. Osteochondromyxoma⁣^∗∗^

Supplemental criteria	1. Affected first-degree relative
	2. Activating pathogenic variants of PRKACA (single base substitutions and copy number variation) and PRKACB (Beuschlein, Fassnacht et al. 2014; Forlino, Vetro et al. 2014)
	3. Inactivating mutation of the PRKAR1A gene (Bossis, Voutetakis et al. 2004)

Minor criteria (findings suggestive of CNC but not diagnostic)	1. Intense freckling (without darkly pigmented spots or typical distribution)
	2. Blue nevus, common type (if multiple)
	3. Café-au-lait spots or other “birthmarks”
	4. Elevated IGF-1 levels, abnormal glucose tolerance test (GTT), or paradoxical GH response to TRH testing without clinical acromegaly
	5. Cardiomyopathy
	6. History of Cushing's syndrome, acromegaly, or sudden death in extended family
	7. Pilonidal sinus
	8. Colonic polyps (usually in association with acromegaly)
	9. Multiple skin tags or other skin lesions; lipomas
	10. Hyperprolactinemia (usually mild and often combined with clinical/subclinical acromegaly)
	11. Single benign thyroid nodule in a child < 18 years; multiple nodules in adults (> 18 years)
	12. Family history of carcinoma (thyroid, colon, pancreas, and ovary); other multiple benign or malignant tumors

**Table 3:** Tumors reported to be associated with the CNC and their approximate frequencies.

**Organ**	**Manifestation**	**%**
Skin	Lentigines	70–80
	Blue nevus	40
	Epitheliod blue nevus	
	Cutaneous myxomas	30–50
	Café-au-lait spots	Rare
	Depigmented lesions	Rare
	Spitz nevus	Rare

Pituitary	Somatomammotroph hyperplasia	67
	Asymptomatic elevation of GH, IGF1 or prolactin	Up to 75
	GH-producing adenoma with acromegaly	10–12
	Prolactinomas	Rare

Eye	Facial and palpebral lentigines	70
	Pigmented lesions of the caruncle or conjunctival	27
	Eyelid myxomas	Rare
	Pigmented schwannomas of the uvea	

Thyroid	Cystic or Nodular disease	Up to 75
	Benign thyroid adenomas	Up to 25
	Thyroid cancer (papillary or follicular type)	Up to 10

Heart	Cardiac myxomas	20–40

Pancreas	Acinar cell carcinoma	
	Adenocarcinoma	2.5
	Intraductal pancreatic mucinous neoplasia (IPMN)	

Liver	Hepatocellular adenoma	Rare

Adrenals	PPNAD	25–60
	Adrenocortical cancer	Rare

Testes	LCCSCT	41
	Leydig-cell tumors	Rare
	Adrenocortical rest tumors	Rare

Ovaries	Ovarian cyst	
	Serus cyst adenomas	14
	Cystic teratomas	

Breast	Breast and nipple myxomas	
	Ductal adenomas	20
	Myxoid fibroadenomas	

Uterus	Uterine myxoid tumors	Rare

Bone	Osteochondromyxoma	Rare

Nerve sheath	PMS	Up to 10

Other organs	Paratiroid mixed tumor	
	Bronchogenic cyst	Rare
	Colonic and gastric carcinoma	
	Peritoneal fibrous histiocytomas	

## Data Availability

The figure and Excel files supporting the findings of this study are available within the article and supporting files.

## Supporting Information

Supporting Information Additional supporting information can be found online in the Supporting Information section. CARE_Checklist_Kobayashi_Updated.docx – Completed CARE checklist in accordance with the CARE guidelines for case reports.
