# Supplementary material for: A Case of Myxoid Malignant Peripheral Nerve Sheath Tumor in a Patient With Carney Complex
Source: Case Rep Pathol. 2025 Jul 31;2025:4337436. doi: 10.1155/crip/4337436 (PMC12331405; doi:10.1155/crip/4337436)
Supplement: Supporting Information — Additional supporting information can be found online in the Supporting Information section. CARE_Checklist_Kobayashi_Updated.docx – Completed CARE checklist in accordance with the CARE guidelines for case reports. [file 4337436.f1.docx]

# CARE Checklist

This CARE Checklist is completed for the manuscript entitled "A case of myxoid malignant peripheral nerve sheath tumor in a patient with Carney complex."

| Checklist Item | Reported on Page/Section |
| --- | --- |
| 1. Title | Yes – Title includes 'case report' |
| 2. Key Words | Yes – 'Carney complex, MPNST, PRKAR1A mutation' |
| 3. Abstract | Yes – Structured abstract provided (Background, Case presentation, Conclusion) |
| 4. Introduction | Yes – Page 1–2, provides background and rationale |
| 5. Patient Information | Yes – Page 2, includes age, sex, medical and family history |
| 6. Clinical Findings | Yes – Page 2–3, includes symptoms and physical findings |
| 7. Timeline | Yes – Page 2–3, chronological events from age 45 to 73 described |
| 8. Diagnostic Assessment | Yes – Page 3–4, includes imaging, pathology, genetic testing, differential diagnosis |
| 9. Therapeutic Intervention | Yes – Page 3–4, multiple surgeries and treatments described |
| 10. Follow-up and Outcomes | Yes – Page 3–4, recurrence and outcome (death at age 73) described |
| 11. Discussion | Yes – Pages 4–6, includes interpretation, literature context, and learning points |
| 12. Patient Perspective | Although the patient passed away before the preparation of this case report, her husband expressed deep gratitude that her long and difficult medical journey would be shared with the medical community. He was moved to tears and said, “Now I can finally tell her that her story will help others.” His response emphasized the importance of documenting and sharing rare cases such as this. |
| 13. Informed Consent | Yes – Page 6, approved by Ethics Committee with number provided |
